# An Herbal Medicine, Yukgunja-Tang is more Effective in a Type of Functional Dyspepsia Categorized by Facial Shape Diagnosis: A Placebo-Controlled, Double-Blind, Randomized Trial

**DOI:** 10.1155/2018/8546357

**Published:** 2018-10-01

**Authors:** Seok-Jae Ko, Jae-Woo Park, Jae-hyung Lee, Jung-eun Lee, Na-yeon Ha, Seong-uk Nam, Jae-hong Lee, Soo-Hyung Jeon, Jong-Won Kim, Changwan Kang, Inkwon Yeo, Jinsung Kim

**Affiliations:** ^1^Department of Gastroenterology, College of Korean Medicine, Kyung Hee University, Kyungheedae-ro 26, Dongdaemun-gu, Seoul 02447, Republic of Korea; ^2^Department of Sasang Constitutional Medicine, College of Korean Medicine, Dong-Eui University, 62 Yangjeong-ro, Busanjin-gu, Busan 47227, Republic of Korea; ^3^Production Information Technology Engineering Major, Dong-Eui University, 62 Yangjeong-ro, Busanjin-gu, Busan 47227, Republic of Korea; ^4^Department of Statistics, Sookmyung Women's University, Cheongpa-ro 47-gil 100, Yongsan-gu, Seoul 140-742, Republic of Korea

## Abstract

**Introduction:**

Functional dyspepsia (FD) is a functional gastrointestinal disorder characterized by persistent upper dyspeptic symptoms without organic lesions. There is no standard therapy for FD. Yukgunja-tang (YGJT) is an herbal medicine used for treating upper gastrointestinal symptoms in Asia. Studies on the effect of YGJT on FD have been conducted. However, the results were inconsistent. In* Hyungsang* medicine, traditional Korean medicine, FD patients are classified into bladder body (BB) or gallbladder body (GB) subtypes by the shape and angle of their faces. Each subtype may have different characteristics, physiology, and pathology of the same disease. YGJT is more effective for patients with BB subtype. The three-dimensional facial shape diagnostic system (3-FSDS) was shown to be effective in diagnosing BB or GB subtypes. This study aimed to investigate the effect of YGJT on FD patients classified using the 3-FSDS.

**Materials and Methods:**

The current study was a placebo-controlled, double-blinded, randomized, two-center trial. Eligible patients were diagnosed with either BB or GB FD subtype using the 3-FSDS. Ninety-six participants (48 BB and 48 GB subtypes) were randomly allocated to treatment or control groups in a 2:1 ratio. YGJT or placebo was administered for eight weeks. The primary outcome was assessed using the total dyspepsia symptom scale (TDS), while the secondary outcomes were assessed using the single dyspepsia symptom scale (SDS), proportion of responders, visual analog scale, Nepean dyspepsia index, functional dyspepsia-related quality of life, and spleen qi deficiency questionnaire.

**Results and Discussion:**

The result of TDS showed the superior effect of YGJT on BB over GB subtype. The subgroup analysis of TDS and SDS scores showed the superior effect of YGJT over placebo. Other outcome variables did not show any significant differences between groups.

**Conclusion:**

YGJT may be considered for FD patients diagnosed with BB subtype using 3-FSDS.

## 1. Introduction

Diagnosis by the shape and appearance of patients historically originated from ancient medical texts (*Huangdi Neijing,* Yellow Emperor's Internal Classic) in Asia and many traditional Korean medicine (TKM) doctors have been using it until now [1]. Based on this diagnostic method, Park et al. developed a unique medicine theory called* Hyungsang* medicine [2]. According to* Hyungsang* medicine, patients are classified into various subtypes such as bladder body (BB) or gallbladder body (GB) based on the shapes of their face and body [3]. Depending on the subtype, the cause and treatment of the disease may vary. For example, patients with BB subtype are more vulnerable to dyspepsia, obesity, and lethargy [4]. They lack yang and qi and produce a lot of dampness and phlegm; therefore, TKM practitioners tend to prescribe drugs to invigorate qi and reduce dampness and phlegm [4, 5]. Considering that* Hyungsang* diagnosis focuses on morphological features of patients, the excellence of TKM in distinguishing individual characteristics is emphasized. However, though it plays an important role for TKM doctors,* Hyungsang* diagnosis mostly depends on individual experience and perspectives of practitioners; therefore, the diagnoses of doctors are often inconsistent, which makes communication difficult among TKM doctors and hinders the advancement of* Hyungsang* medicine. In order to overcome these limitations, research on standardization and objectification of diagnosis by the appearance of patients has been actively conducted [6–8]. As a result, questionnaires, three-dimensional (3D) body measuring machines, and 3D face automatic recognizers have been developed [9–12]. A 3D facial shape diagnostic system (3-FSDS) consists of a 3D diagnostic scanner combined with a face 3D scanner, data acquisition program, and facial shape measurement and diagnostic program. Three-dimensional stereoscopic images of the frontal and temporal faces are acquired, and 3D coordinates are obtained to calculate distance, angle, and area. A significant variable is selected from the results after calculation, and BB or GB subtype is diagnosed. The 3-FSDS proved to be effective in diagnosing BB/GB subtypes in a clinical trial for the approval of the Korea Food and Drug Administration (KFDA), and its diagnostic rate was over 70%. In 2011, the device was approved by the KFDA (No. 11-500 Medical Image Analysis Apparatus, May 4, 2011).

Functional dyspepsia (FD) is a common functional gastrointestinal disorder characterized by persistent or recurrent abdominal pain, discomfort, and other dyspeptic symptoms in the absence of organic diseases [13]. FD has a prevalence of approximately 11-25% in the total population, and about 50% of patients with FD complain of dyspepsia without any structural disease [14, 15]. Recently in the United Kingdom (UK), annual medical costs for FD have been reported to be more than one billion pounds, and the huge economic burden caused by FD has a considerable negative impact on the society [16, 17]. Several causative factors are involved in FD such as gastrointestinal motility disorder, sensory disturbance, and* H. pylori* infection, and they tend to interact with each other [18, 19]. Proton-Pump inhibitors, prokinetics, and dietary modifications are prescribed as western medical treatments [20]. However, due to unsatisfactory response to conventional treatments, patients are turning to alternative and complementary medicine such as herbal medicines [21, 22].

Yukgunja-tang (YGJT;* Rikkunshito* in Kampo Medicine;* Liu Jun Zi Tang* in Traditional Chinese Medicine) is an herbal medicine made up of eight crude herbs that have been used for treating upper gastrointestinal symptoms such as indigestion, abdominal pain, and epigastric discomfort in Asia [23, 24]. YGJT has been reported to maintain gastric storage capacity and facilitate stomach emptying leading to improved gastric accommodation [25, 26]. Several clinical studies also demonstrated the ameliorative effect of YGJT in FD, and a recent systematic review and meta-analysis reported that YGJT might be more effective compared to prokinetic drugs in the treatment of functional dyspepsia without any side effects [27, 28]. YGJT is known to be more effective for improving dyspeptic symptoms in patients with the BB subtype of FD [4, 5].

In this study, we classified FD patients, using 3-FSDS, into one of two subtypes based on the* Hyungsang* diagnosis: BB or GB subtype. After diagnosis, either YGJT or a placebo was administered for eight weeks and the effect of YGJT was compared. The aim of this study is to investigate the efficacy of YGJT on FD patients diagnosed with either BB or GB subtype by 3-FSDS. Through this study, the conventional* Hyungsang* theory that YGJT has superior effect on the BB subtype compared to the GB subtype of FD will be confirmed, and the usefulness of 3-FSDS will be verified.

## 2. Materials and Methods

### 2.1. Study Protocol

The current study was a placebo-controlled, double-blinded, randomized, two-center trial. After screening tests, eligible patients were diagnosed with either BB or GB subtypes of FD using the 3-FSDS. A total of 96 participants (48 BB and 48 GB subtypes) were recruited, and patients with each subtype were randomly allocated to treatment or control groups in a 2:1 ratio by an independent statistician. The number of patients in each group was similar to the number of patients who received placebo YGJT. The reason for the 2:1 ratio random allocation was that it would be unethical to assign an equal number of FD patients to the ineffective placebo treatment group [29]. The participants completed a one-week run-in (weeks −1 to 0), followed by eight weeks of either YGJT or placebo administration (weeks 0–8). The 8-week treatment duration was based on guideline for ROME III criteria [30]. During the administration period, participants were supposed to take one pack of YGJT or its placebo three times a day (1 h after each meal). The trial protocol was approved by the Institutional Review Board of the Kyung Hee University Korean Medicine Hospital (IRB number KOMCIRB-160115-HR-001) and the Dong-Eui University Korean Medicine Hospital (IRB number 2016-01). The protocol for this study was previously published [31] and there were no major changes to the design after initiation of the study.

### 2.2. Participants

Participants who met the criteria for FD based on Rome III [13] were recruited at two different sites in Korea: the Kyung Hee University Korean Medicine Hospital and the Dong-Eui University Korean Medicine Hospital. The inclusion and exclusion criteria for this study are shown in Table 1. Informed consent was obtained prior to the start of the trial, and participants were free to withdraw from the study at any time. This study was conducted between February 2016 and April 2018.

### 2.3. Randomization and Blinding

Randomization was performed with the PROC PLAN of SAS 9.4 (SAS Institute Inc., Cary, NC, USA) by the independent statistician. Eligible patients were assessed using the 3-FDSD and classified into either BB or GB subtypes. Each patient was assigned a randomization number according to a predetermined allocation list at each center. Patients were sequentially assigned to either treatment or placebo groups in a 2:1 ratio, and if any of the two groups containing either the BB or GB subtypes was full, patients in such groups were excluded from the study thereafter. To preserve allocation concealment, we used an opaque envelope containing randomization code. Only the independent statistician was not blinded to the randomization. Other researchers associated with the study (including subjects, investigators, clinical research coordinator, and clinical pharmacist) were blinded to the random allocation.

### 2.4. Three-Dimensional Facial Shape Diagnostic System (3-FSDS)

**Figure 1:**
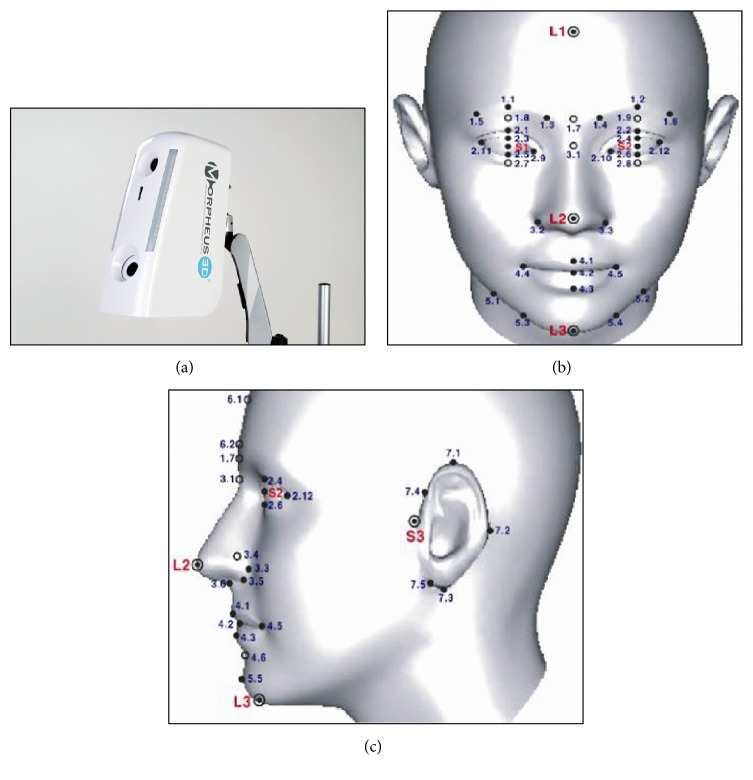
(a) 3-Dimensional facial shape diagnostic system (3-FSDS), (b) the 3D coordinates of frontal face used in 3-FSDS, (c) the 3D coordinates of side face used in 3-FSDS.

The 3-FSDS is composed of a 3D facial scanner (Morpheus 3D), scanner driving and data generation program (Real Face), 3D facial shape measurement program (Renai MEF), and 3D facial shape diagnosis program (Renai FSD) (Figure 1(a)). The 3-FSDS photographs the front and side faces of subjects as a 3-dimensional image and obtains the 3D coordinates of 39 measurement points from the frontal face and 15 measurement points from the side face (Figures 1(b) and 1(c)). The distance and angular area between the points are calculated to generate 337 variables. After stepwise analysis among variables, significant variables are selected and either the BB or GB subtype is determined. For example, the length of the face can be calculated from the distance between the forehead (Figure 1(b) L1) and the jaw (Figure 1(b) L3), and the lateral width is calculated from the distance from the ear (Figure 1(c) S3) to the midpoint between both eyes (Figure 1(c) point 3.1). The width of the front side is calculated from the distance between the left and right eyebrow end points. Because the side face is more developed than the front one in the BB subtype, the area of the side face is larger than that of the front face. In the GB subtype, the area of the side face is smaller than that of the front face. A detailed explanation of the 3-FSDS was described in a previously published paper [31].

### 2.5. Interventions

Patients were supposed to take one pack (5 g) of YGJT or placebo three times a day for eight weeks (administration period). The YGJT used in the trial was a brown, bitter, herbal extract granule (Yukgunjatang granule®, Hankookshinyak Co., Ltd., Nonsan, Korea) and was produced according to the Korean Good Manufacturing Practice. Yukgunjatang granule®, a water-extracted YGJT combined with starch and lactose, was approved by the Korean Food & Drug Administration. One pack of YGJT is composed of the eight herbal plants listed in Table 2. Placebo YGJT consisted primarily of cornstarch powder with a similar color and tastes similarly to the real YGJT. The real YGJT and its placebo were identically packed and sealed in similar opaque aluminum bags with similar labeling. The patients were supposed to return the unused YGJT or placebo, and the treatment compliance was calculated by subtracting the remaining drugs from the planned drugs and dividing the resulting value by the planned drugs.

### 2.6. Outcome Assessments

#### 2.6.1. Primary Outcome

*Total Dyspepsia Symptom Scale (TDS Scale)*. The TDS scale is composed of eight items (postprandial fullness and bloating, early satiety, epigastric pain, epigastric burning, nausea, vomiting, and belching), with a four-point Likert scale [29, 32]. The TDS score is obtained from the sum of the scores of the eight items. This scale was investigated at baseline and after four and eight weeks.

#### 2.6.2. Secondary Outcomes

*(1) Single Dyspepsia Symptom Scale (SDS Scale)*. The SDS scale comprises three properties (frequency, intensity, and level of discomfort) of four major symptoms (epigastric pain, epigastric burning, postprandial fullness and bloating, and early satiety) of FD with a 4-point Likert scale [29, 32]. The SDS score is the sum of the three properties of the four symptoms. The SDS scale was evaluated at baseline and after four and eight weeks.

*(2) Proportion of Responders (PR) for FD Pain and Discomfort*. PR was defined as the proportion of patients with at least 50% reduction in FD pain and discomfort from week 0 to week 8. PR was assessed using a weekly adequate relief (AR) question: “In the past 7 days, have you had adequate relief of your pain or discomfort related FD?” AR was assessed during visits or over the telephone. AR and PR have been effective for assessing improvement in abdominal pain and discomfort of functional gastrointestinal diseases [26, 33].

*(3) Visual Analogue Scale (VAS)*. The VAS measures the severity of overall dyspeptic symptoms on a 100-mm visual analog scale during the entire trial (ranging from 0 mm, which signifies no symptom, to 100 mm, which signifies the most severe symptom ever experienced). The VAS was evaluated at baseline and after four and eight weeks.

*(4) Nepean Dyspepsia Index-Korean Version (NDI-K)*. The Nepean dyspepsia index (NDI) developed by Talley et al. was reported to be a reliable questionnaire for measuring dyspeptic symptoms and health-related quality of life [34, 35]. The Korean version of the NDI (NDI-K) was validated by Lee et al. and has been used in clinical studies [9, 36]. We used symptom-based questions about the period, severity, and degree of distress of 15 symptoms with a 5- or 6-point Likert scale at baseline and after four and eight weeks.

*(5) Functional Dyspepsia-Related Quality of Life (FD-QoL) Questionnaire*. The FD-QoL questionnaire comprises four sections with a total of 21 questions regarding quality of life measured by a 5-point Likert scale (0: not at all or not applicable, 1: a little, 2: moderately, 3: quite a lot, and 4: extremely). The four sections comprise questions on diet (5 items), daily activity (4 items), emotion (6 items), and social functioning (6 items). The FD-QoL questionnaire is known to be reliable for assessing the overall quality of life of patients [37]. The FD-QoL questionnaire was evaluated at baseline and after four and eight weeks.

*(6) Spleen Qi Deficiency Questionnaire (SQDQ)*. The SQDQ has been used to assess spleen qi deficiency syndrome, which is the most common symptom in FD patients [38, 39]. The questionnaire developed by Oh et al. [40] is composed of 11 items, and the total score is the sum of the score of each weighed item. The cut-off value of the SQDQ to determine spleen qi deficiency syndrome is 43.18 [41]. The SQDQ was measured at baseline and after four and eight weeks.

### 2.7. Safety and Adverse Events

To select eligible subjects and to confirm the safety of administering YGJT for eight weeks, blood biochemical tests (measurement of white blood cell count, red blood cell count, hemoglobin, hematocrit, platelet, aspartate aminotransferase, alanine aminotransferase, gamma-glutamyl transpeptidase, blood urea nitrogen, creatinine, and erythrocyte sedimentation rate) were performed before randomization and after treatment was completed. Adverse events (AEs) were reported and documented in detail during the entire study period. Serious AEs were promptly reported to the institutional review board and the principal investigator within 24 hours.

### 2.8. Sample Size Calculation

The sample size was calculated according to the following formula:

(1) N=nt+ncnt  is the number of treatment groups; nc is the number of control groups.nc=12nt=Zα/2+Zβ2σ2λ+1/λμc−μt2

A previous study reported 1.57 points of improvement (*μ*_c_ − *μ*_t_ = *δ*) in the TDS scale for FD after four weeks of YGJT administration compared to placebo [29]. A mean standard deviation (SD = *σ*) of 2.148 was obtained. The ratio of the treatment group to the control group in the present study was 2:1; therefore the ratio (*λ*) was 2. With a power (1 − *β*) of 80% and a significance level (*α*) of 5%, assuming *δ* = 1.57 and *σ* = 2.148, a sample size of n_t_ = 44 and n_c_ = 22 subjects was calculated. Considering an assumed dropout rate of 30%, a total of 96 subjects were required.

### 2.9. Statistical Analysis

All data were collected and handled by an independent statistician. Other researchers associated with the study (including clinicians, clinical research coordinator, and clinical pharmacist) were rigorously isolated from data collection and analysis until the last participant completed the trial. Both the intention-to-treat (ITT) using the baseline observation carried forward approach for missing data and per-protocol (PP) populations were analyzed. All data are presented as mean ± standard deviation or number (%). Baseline characteristics between patients were analyzed using the chi-squared or Fisher's exact test for categorical variables and analysis of variance (ANOVA) for continuous variables. For the efficacy analysis, one-way ANOVA with Dunnett's test as a post hoc test was performed to compare changes in the scores of each outcome for eight weeks between the intervention groups or the facial shape types. Multiple comparisons of nonparametric data were performed using the Kruskal-Wallis* H* test followed by Dunnett's T3 post hoc test. For analysis between two subgroups, we used the independent two-sample* t*-test or Mann–Whitney* U* test as nonparametric statistical tests. Efficacy was analyzed based on the change rate variable which is defined as the value obtained by subtracting the value at the end of eight weeks from the value at baseline, and dividing it by the value at baseline. All statistical analyses of the data were performed using the SPSS software, version 20.0 (IBM SPSS Statistics, New York, USA), and a* P* value < 0.05 was regarded as statistically significant.

## 3. Results

### 3.1. Demographic Characteristics and Baseline Symptoms

**Figure 2:**
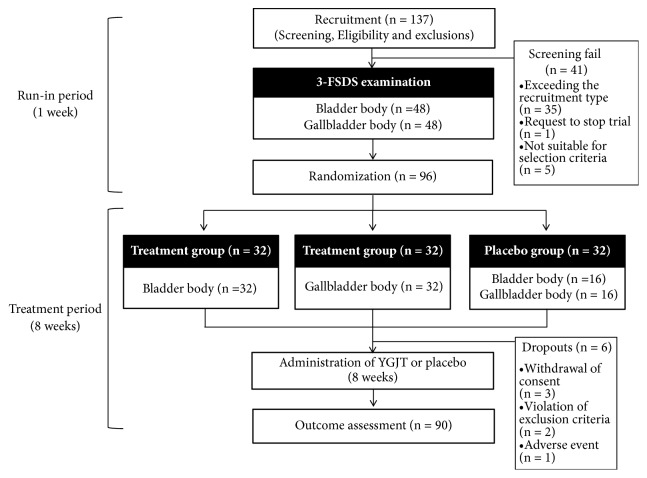
Flow chart of the trial. 3-FSDS, 3-dimensional facial shape diagnostic system; YGJT, Yukgunja-tang.

A total of 137 patients were screened. Of these, 41 participants did not meet the screening criteria (Figure 2). Ninety-six patients were enrolled and randomly assigned to either the treatment or placebo group. Ninety participants completed the study and six patients were dropped from the study due to withdrawal of consent, violation of exclusion criteria, and adverse events (Figure 2). Baseline demographic characteristics, TDS scale, SDS scale, VAS for overall dyspeptic symptoms, NDI-K, FD-QoL questionnaire, and SQDQ were well balanced among groups at the beginning of the study (Table 3).

### 3.2. Primary Outcome

**Figure 3:**
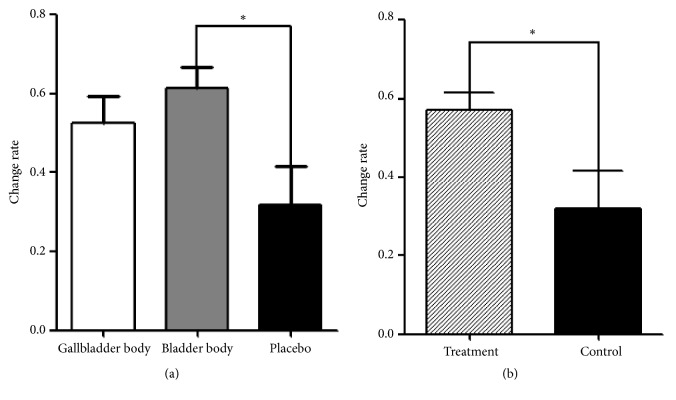
(a) Comparison of change rate of Total Dyspepsia Symptom (TDS) scale among 3 groups. Analysis was performed by one-way ANOVA with Dunnett T3 post hoc test. *∗P* < 0.05. (b) Comparison of change rate of TDS scale between treatment and control group as subgroup analysis. Analysis was performed by Mann–Whitney* U* test. *∗P* < 0.05.

After the treatment period, the score on the TDS scale was significantly different among three groups (Table 4), and only the group comprising the BB subtype showed significant improvement compared to the placebo group according to the post hoc test (Figure 3(a)). After subgroup analysis between treatment and placebo groups, the TDS scale scores showed significant superior effect of real YGJT compared to placebo (Table 5 and Figure 3(b)).

### 3.3. Secondary Outcomes

There was no significant difference among the three groups based on the score on the SDS scale, VAS for overall dyspeptic symptoms, NDI-K, FD-QoL questionnaire, SQDQ, and PR (Table 4). Only the SDS scale score among the secondary outcomes displayed significant improvement in the treatment group compared to the placebo group according to subgroup analysis (Table 5).

### 3.4. Adverse Events and Treatment Compliance

One major adverse event occurred during this study. During the treatment period, one patient underwent routine annual health check-ups and brain tumor was suspected through brain-MRI examination. During brain biopsy, for further evaluation, intracerebral hemorrhage occurred and the patient was admitted to the intensive care unit after an emergency operation. We reported an adverse event immediately to the institutional review board and the principal investigator within 24 hours and documented it in detail. We also carried out regular follow-up investigations on the patient, and the adverse event proved to be “definitely not related” to the study YGJT or placebo. The treatment compliance of treatment group was 89.16% and that of placebo group was 87.44%. Total treatment compliance was calculated as 88.58%.

## 4. Discussions

This study aimed to investigate the efficacy of YGJT on different types of FD patients classified by 3-FSDS. This study also aimed to verify the clinical usage of 3-FSDS as a diagnostic tool. The result of TDS as a primary outcome in this study showed the superior effect of YGJT on BB over GB subtype of FD. The subgroup analysis of the TDS and SDS scores also showed the superior effect of YGJT compared to placebo regardless of subtypes of FD patients. Other outcome variables did not show any significant difference between the FD groups.

The significant effect of YGJT on FD has been demonstrated by a number of experimental and clinical studies [42, 43]. Basic studies showed that YGJT attenuated gastric dysmotility induced by a nitric oxide-synthesizing enzyme inhibitor and improved the delay of gastric emptying mediated by serotonin (5-HT) type 3 receptor [26, 44]. Conversely, YGJT increased plasma ghrelin level that has been known to have a strong orexigenic effect and enhancement of GI motility [45–48]. Clinically, YGJT may improve stress-induced gastric hypersensitivity and/or changes in gastric wall tone detected by gastric barostat method [49]. In addition, YGJT reversed the increase in plasma levels of neuropeptide Y, a representative neurotransmitter of the sympathetic nervous system, leading to improvement of the dyspeptic symptoms [50]. However, recent studies on YGJT did not show significant differences compared to the placebo in specific variables assessing upper dyspeptic symptoms such as epigastric burning, postprandial fullness, and early satiation [51, 52]. These inconsistent results might be due to reasons including small sample size, regression to the mean effect, or high placebo effect. From the results of this study, the differential effect of YGJT depending on subtypes of FD patients might be one of the major reasons for the insignificant effect of YGJT. YGJT, as an herbal medicine based on TKM theory, was prescribed after pattern identification in TKM diagnosis [29]. For example, a recent randomized controlled trial reported that YGJT appears to offer symptomatic improvement in FD patients with spleen-deficiency and qi-stagnation syndrome [29], and studies on* Hyungsang* medicine demonstrated that YGJT can improve FD symptoms for patients with BB subtype of FD [5]. Patients with BB and GB subtypes of FD have different characteristics, appearances, and pathological and physiological features for the same disease [3]. Patients with the BB subtype of FD tend to have deficient qi and excessive dampness. Therefore, they are susceptible to obesity, general weakness, joint disease, and narcolepsy [4]. On the other hand, patients with the GB subtype of FD are easily irritated, emotionally anxious, sleep deprived, and anemic, because they have deficient yin-blood and excessive heat [4]. YGJT is known to invigorate and eliminate dampness; therefore, it is a more appropriate prescription for patients with the BB subtype of FD [29].

We chose FD as the target disease to verify the usefulness of 3-FSDS for classifying either BB or GB subtypes. FD affects a large number of patients in the TKM field and the number of patients with FD is constantly increasing [53]. In* Hyungsang* medicine, which is part of TKM, functional gastrointestinal disease is the second most prevalent after metabolic disease [54]. There are currently no satisfactory standardized treatments for FD. TKM can play an important role in the treatment of diseases without organic causes such as FD. FD is a functional disease in which the efficacy of therapeutic medication varies according to the type of diagnosis. YGJT is a representative herbal prescription for FD in TKM.

There are several limitations in this study. First, 3-FSDS has a diagnosis rate of 70-80% and cannot distinguish between BB and GB subtypes completely. Therefore, there might be patients in the gray zone between BB and GB subtypes who affect the results of this study. Future studies with a large sample size may be a solution for this limitation. Second, other variables that affect classification into either BB or GB subtypes such as face area, face color, personality, and size of body could be considered in future studies. This would make it possible to improve diagnosis using 3-FSDS and obtain a higher diagnosis rate. Third, one reason for the insignificant results between groups of secondary outcomes might be that there are too many patients with mild levels of symptom severity. Functional diseases like FD are well known to be highly affected by placebos; therefore medications for FD might have more definite effect in patients with moderate or severe dyspeptic symptoms which might be observed during long-term follow-up. Lastly, FD can be classified into subtypes including epigastric pain and postprandial distress syndromes according to the existing Rome criteria. Further studies on the relationship between the classification of FD using the Rome criteria and* Hyungsang* medicine are needed.

## 5. Conclusions

This study was the first to classify FD patients into different subtypes using* Hyungsang* medicine and to compare the effect of YGJT between subtypes. In the study, YGJT was more effective compared to the placebo, especially in FD patients diagnosed with BB subtype using 3-FSDS. In addition, 3-FSDS has been identified as a useful diagnostic tool for distinguishing between FD subtypes in* Hyungsang* medicine. In the present study, there were no significant differences in most secondary outcomes; therefore, further studies with a larger sample size are needed, and other variables affecting diagnosis rate should be considered in future studies.

## Figures and Tables

**Table 1:** Inclusion and exclusion criteria of the study.

Inclusion criteria	(1) Subjects aged 19–75 years
	(2) Subjects who meet the Rome III criteria for functional dyspepsia
	(3) Subjects with more than 40 points on the visual analog scale (VAS; 0, no discomfort; 100, most severe discomfort) for the severity of dyspeptic symptoms
	(4) Subjects who agree to receive no other treatments during the study
	(5) Subjects who voluntarily agree with the study protocol and sign a written informed consent

Exclusion criteria	(1) Subjects with peptic ulcer or gastroesophageal reflux disease confirmed on esophagogastroduodenoscopy
	(2) Subjects with obvious signs of irritable bowel syndrome
	(3) Subjects with alarm symptoms, such as severe weight loss, melena, and dysphagia
	(4) Subjects with severe systemic organ diseases (cancer, diseases of heart, lung, liver, or kidney) or mental illness
	(5) Subjects who have had surgery related to the gastrointestinal tract, except for appendectomy more than six months ago
	(6) Subjects taking drugs that might affect the gastrointestinal tract; a minimum wash-out period of a week is required before participating in the study
	(7) Subjects who have had maxillofacial surgery or facial bone contouring surgery
	(8) Subjects who are pregnant or breastfeeding
	(9) Subjects who have malabsorption or maldigestion
	(10) Human immunodeficiency virus (HIV) positive subjects
	(11) Subjects with difficulties in taking part in the study (e.g., serious mental illness, dementia, drug addiction, time constraint, severe disorder in vision or hearing, illiteracy)
	(12) Subjects who have taken investigational drugs for other trials in the last three months

**Table 2:** Herbal plants contained in Yukgunja-tang granule.

**Scientific name**	**Dosage per pack (gram)**
*Pinelliae tuber*	1.33
*Citriunshii pericarpium*	1.33
*Ginseng radix alba*	1.33
*Atractylodis rhizoma alba*	1.33
*Hoelen*	1.33
*Glycyrrhizae radix*	0.50
*Zingiberis rhizoma crudus*	0.67
*Zizyphi fructus*	0.67

**Table 3:** Characteristics of the patients and baseline TDS scale, SDS scale, VAS for overall dyspeptic symptoms, NDI-K, FD-QoL questionnaire, and SQDQ.

Variables/Group	Gallbladder body	Bladder body	Placebo	*P* value
	(*n* = 32)	(*n* = 32)	(*n* = 32)	
Mean age	47.16 (11.83)	47.22 (11.60)	45.28 (12.27)	0.761
Mean weight (kg)	56.56 (7.93)	60.01 (9.39)	57.60 (10.28)	0.313
Mean BMI (kg/m^2^)	21.98 (2.85)	23.28 (3.09)	22.21 (2.74)	0.165
Male (%)	15.6	15.6	15.6	1.000

TDS scale	8.81 (3.60)	8.41 (3.09)	8.66 (4.35)	0.907
SDS scale	15.28 (5.76)	14.78 (6.63)	14.72 (6.61)	0.927
VAS for overall dyspeptic symptoms	68.03 (12.09)	60.75 (18.24)	63.16 (9.06)	0.101
NDI-K	52.28 (27.74)	49.63 (30.48)	51.41 (30.56)	0.935
FD-QoL questionnaire	28.44 (20.98)	19.28 (11.77)	25.09 (17.80)	0.106
SQDQ	47.97 (19.22)	39.21 (15.50)	47.16 (16.15)	0.081

TDS: Total Dyspepsia Symptom; SDS: Single Dyspepsia Symptom; VAS: Visual Analogue Scale; NDI-K: Nepean Dyspepsia Index-Korean Version; FD-QoL: Functional Dyspepsia-Related Quality of Life; SQDQ: Spleen Qi Deficiency Questionnaire; BMI: Body Mass Index.

Both gallbladder and bladder body groups are treatment groups.

Baseline values were analyzed by Pearson's chi-squared test for categorical variables and one way-ANOVA for continuous variables.

Continuous variables are presented as mean (standard deviation).

*P* value < 0.05 is regarded as statistically significant.

**Table 4:** Change rate of TDS scale, SDS scale, VAS for overall dyspeptic symptoms, NDI-K, FD-QoL questionnaire, SQDQ, and proportion of responders.

Change rate of variables/Group	Gallbladder body	Bladder body	Placebo	*P* value
	(*n* = 32)	(*n* = 32)	(*n* = 32)	
TDS scale	0.53 (0.38)	0.61 (0.31)	0.32 (0.54)	0.038*∗*
SDS scale	0.55 (0.36)	0.57 (0.43)	0.34 (0.72)	0.163
VAS for overall dyspeptic symptoms	0.43 (0.25)	0.48 (0.38)	0.41 (0.28)	0.692
NDI-K	0.57 (0.35)	0.61 (0.42)	0.44 (0.49)	0.274
FD-QoL questionnaire	0.58 (0.40)	0.64 (0.31)	0.19 (2.20)	0.338
SQDQ	0.36 (0.35)	0.44 (0.33)	0.34 (0.36)	0.457
Proportion of responders (%)	87.1	78.1	74.2	0.431

TDS: Total Dyspepsia Symptom; SDS: Single Dyspepsia Symptom; VAS: Visual Analogue Scale; NDI-K: Nepean Dyspepsia Index-Korean Version; FD-QoL: Functional Dyspepsia-Related Quality of Life; SQDQ: Spleen Qi Deficiency Questionnaire.

Both gallbladder and bladder body groups are treatment groups.

Change rate is defined as the value obtained by subtracting the value of 8 weeks from the value of baseline and dividing it by the value of baseline.

Values were analyzed by Pearson's chi-squared test for categorical variables and one way-ANOVA for continuous variables.

Continuous variables are presented as mean (standard deviation).

*∗P* value < 0.05 is regarded as statistically significant.

**Table 5:** Change rate of TDS scale, SDS scale, VAS for overall dyspeptic symptoms, NDI-K, FD-QoL questionnaire, SQDQ, and proportion of responder between treatment and placebo group as subgroup analysis.

Change rate of variables/Group	Treatment	Placebo	*P* value
	(*n* = 64)	(*n* = 32)	
TDS scale	0.57 (0.35)	0.32 (0.54)	0.031*∗*
SDS scale	0.56 (0.39)	0.34 (0.72)	0.046*∗*
VAS for overall dyspeptic symptoms	0.46 (0.32)	0.41 (0.28)	0.483
NDI-K	0.59 (0.39)	0.44 (0.49)	0.135
FD-QoL questionnaire	0.61 (0.35)	0.19 (2.20)	0.463
SQDQ	0.40 (0.34)	0.34 (0.36)	0.402
Proportion of responder (%)	82.5	74.2	0.415

TDS: Total Dyspepsia Symptom; SDS: Single Dyspepsia Symptom; VAS: Visual Analogue Scale; NDI-K: Nepean Dyspepsia Index-Korean Version; FD-QoL: Functional Dyspepsia-Related Quality of Life; SQDQ: Spleen Qi Deficiency Questionnaire.

Treatment group includes gallbladder and bladder body groups.

Change rate is defined as the value obtained by subtracting the value of 8 weeks from the value of baseline and dividing it by the value of baseline.

Values were analyzed by Pearson's chi-squared test for categorical variables and independent two-sample *t*-test as parametric statistical test or Mann–Whitney *U* test as nonparametric statistical test for continuous variables.

Continuous variables are presented as mean (standard deviation).

*∗P* value < 0.05 is regarded as statistically significant.

## Data Availability

The data used to support the findings of this study are included within the article.
